# P-1604. Differential Impact of SARS-CoV-2 Variants of Concern (VOC) on Maternal and Neonatal Outcomes

**DOI:** 10.1093/ofid/ofaf695.1783

**Published:** 2026-01-11

**Authors:** Syeda Fatima Shariq, Adeel A Butt, Atika Jabeen, Muzna Lone, Jameela Ali Al Ajmi

**Affiliations:** Hamad Medical Corporation, Doha, Ad Dawhah, Qatar; Hackensack Meridian JFK University Medical Center , Edison, NJ; Hamad Medical Corporation, Doha, Ad Dawhah, Qatar; Hamad Medical Corporation, Doha, Ad Dawhah, Qatar; Hamad Medical Corporation, Doha, Ad Dawhah, Qatar

## Abstract

**Background:**

SARS-CoV-2 variants have had a differential impact on severity of COVID-19 illness, maternal and neonatal outcomes. Multiple factors contribute to these differences, including strain virulence, local clinical practices, and vaccine effectiveness. To understand variant-specific risks, we determined the differential impact of SARS-CoV-2 variants of concern (VOCs) on maternal and neonatal outcomes.

**Table 1:**
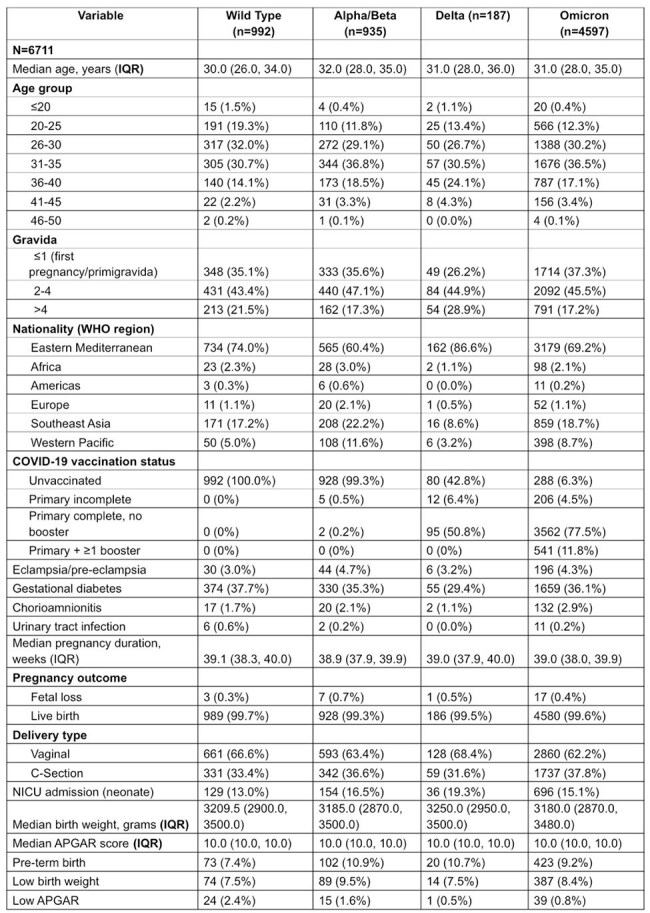
Baseline Characterisitics by COVID-19 Variants of Concern (VOCs)

**Table 2:**
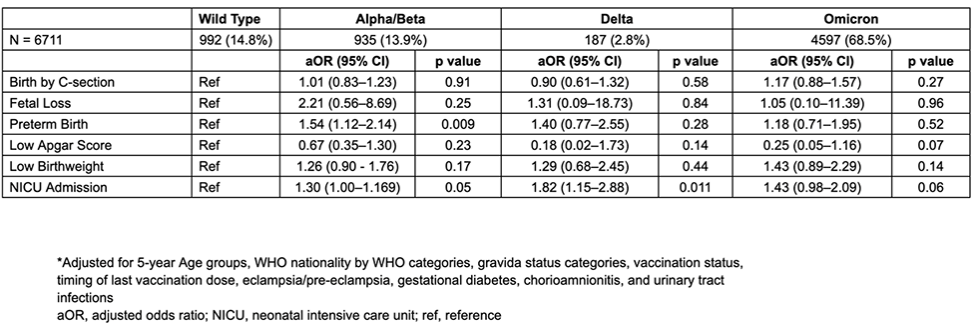
Association of Adverse Maternal and Neonatal Outcomes with SARS-CoV-2 Variants of Concern (VOC) in COVID-19 positive women*

**Methods:**

We conducted a retrospective cohort study at Hamad Medical Corporation (HMC), Qatar, which manages 85% of obstetric cases in the country. Data for pregnant women presenting to HMC (Jan 1, 2020 - May 31, 2024), were retrieved from medical records. We identified women with singleton pregnancies with a first COVID-19 positive test in pregnancy. Those not tested in pregnancy and multiple pregnancies were excluded. The variant of concern (VOC) was approximated by the time of infection, in which Qatar experienced a predominant circulating strain. This approach, used in prior publications, reasonably reflects the infecting VOC at the time. Outcomes included birth by c-section, fetal loss, preterm birth, low Apgar (< 6), low birth weight (LBW < 2500 grams), and neonatal intensive care unit (NICU) admission. Multivariate logistic regression models were used to calculate adjusted odds ratios (aOR).

**Results:**

Among 6,711 COVID-19 positive pregnant women identified, 14.8% were infected in the wild type, 13.9% in Alpha/Beta, 2.8% in Delta, and 68.5% in the Omicron/post-Omicron periods. Infection during the Alpha/Beta period was associated with higher odds of preterm birth (aOR 1.54, 95% CI 1.12–2.14, p < 0.05) compared to wild type period. Infections during the Alpha/Beta and Delta periods were associated with higher odds of NICU admission (Alpha/Beta: aOR 1.30, 95% CI 1.00–1.69, p=0.05; Delta: aOR 1.82, 95% CI 1.15–2.88, p < 0.05). No significant differences were found among variants regarding fetal loss, cesarean delivery, LBW, or low Apgar scores.

**Conclusion:**

COVID-19 infection during the Alpha/Beta and Delta predominant periods was associated with adverse maternal and neonatal outcomes, specifically preterm birth and NICU admissions, compared to wild type period. No significant differences were observed in fetal loss, c-section, LBW, or low Apgar scores during various VOC periods.

**Disclosures:**

All Authors: No reported disclosures

